# The Impact of COVID-19 on Healthcare Services, Risk Management, and Infection Prevention in Surgical Settings: A Qualitative Study

**DOI:** 10.3390/healthcare13060579

**Published:** 2025-03-07

**Authors:** Alice Yip, Jeff Yip, Zoe Tsui, Cheung-Hai Yip, Hau-Ling Lung, Kam-Yee Shit, Rachel Yip

**Affiliations:** 1 S.K. Yee School of Health Sciences, Saint Francis University, 2 Chui Ling Lane, Tseung Kwan O, New Territories, Hong Kong, China; ztsui@sfu.edu.hk (Z.T.); kmyip@sfu.edu.hk (R.Y.); 2 Hong Kong Institute of Paramedicine, Hong Kong, China; jeffreyycyip@gmail.com; 3 Department of Anesthesiology & Operating Suite, Alice Ho Miu Ling Nethersole Hospital, Hong Kong, China; queeniechyip@netscape.net; 4 Department of Anesthesia & Operating Theatre, Tseung Kwan O Hospital, Hong Kong, China; elainelung04@yahoo.com.hk; 5 Department of Surgery, Prince of Wale Hospital, Hong Kong, China; sky366@ha.org.hk

**Keywords:** healthcare professionals, surgical settings, nursing practices, surgical site, surgical infection

## Abstract

**Background/Objective** In every surgical environment, the prevention of surgical site infections is not merely desirable but essential, given their profound impact on patient health and healthcare costs. To optimize patient care during surgery, a thorough exploration and assessment of all intraoperative nursing practices are necessary, guided by empirical evidence. The aim of this study was to explore nurses’ experiences with surgical site infection prevention practices in the intraoperative setting. **Methods** Twenty-one nurses working in clinical settings in Hong Kong participated in semi-structured interviews for this qualitative study. Data were analyzed using Colaizzi’s seven-step method. **Results** Four main themes were identified from the interview data: ensuring safety and minimizing threats; facing silent, intangible, and hidden risks; team collaboration in eliminating risks; and persistent knowledge acquisition. **Conclusions** Nurses encountered diverse obstacles tied to teamwork, updated knowledge, communication, and patient safety. Enhanced quality of care in clinical settings can be achieved through strategic implementations. Focusing on quality improvement initiatives, establishing consistent teams, and recognizing the vital role of nurses strengthen care delivery. These actions contribute significantly to preventing surgical site infections and ensuring patient safety during intraoperative nursing practices.

## 1. Introduction

Infections acquired during surgical procedures contribute to both unnecessary patient morbidity and excessive societal healthcare costs. Consequently, preventing surgical site infections is essential for healthcare providers in every surgical setting [1,2,3]. According to Monahan et al. (2020), low- and middle-income countries experience a greater burden of surgical site infections. Managing these infections can cost between USD 174 and USD 29,610 in these countries, compared to a range of USD 21 to USD 34,000 in high-income European nations [4]. High-income countries also experience significant rates of surgical site infections [4,5]. Improving prevention techniques in clinical settings is an ongoing priority for healthcare providers internationally.

The development of surgical site infections within 1-month post-operation highlights the necessity of rigorous post-operative care and infection control [6]. For certain situations, like following orthopedic joint procedures, surgical site infections manifest at a delayed stage. The primary hazard for contracting infections arises from pathogens infiltrating the surgical wounds [1,7]. The occurrence of surgical site infections can be attributed to factors such as bacterial and virulence load and the ability of the patient to resist infections [7,8]. Lack of knowledge regarding the risk of resistance has caused the rapid growth of antibiotic resistance [9,10]. Previous studies have demonstrated the substantial economic impact of surgical site infections worldwide [3,11]. Low-income countries are disproportionately affected by the ongoing damage caused by antibiotic resistance [12,13].

The reduction of bacterial burden within surgical settings is most important to minimizing the occurrence of surgical site infections during intraoperative procedures. Compliance with evidence-based procedures plays a significant role in achieving this objective. To that end, implementing strict protocols, such as ensuring adherence to hygiene practices by all surgical team members and correct sterilization of operating rooms between procedures, represents a direct approach to lessening the bacterial load [6,14]. While these measures are theoretically effective, maintaining consistent compliance within clinical practice presents an ongoing challenge for healthcare providers. Furthermore, a stronger body of evidence is needed to comprehensively illuminate the impact of surgical site infections on patient outcomes and to further refine preventative strategies [2,15]. Given increasing bacterial antibiotic resistance, preventing surgical site infections is a complex undertaking. It is crucial to reduce operating room microbial contamination to the absolute minimum.

Studies on the prevention and elimination of surgical site infections are vital for creating the best possible operating room conditions and improving patient recovery and well-being [16]. To establish an evidence-based best practice, each facet of intraoperative care must be scrutinized and appraised. Several pre-operative interventions contribute significantly to surgical site infection prevention. These include measures like MRSA screening and decolonization, hair removal (clipping rather than shaving), maintaining normothermia, optimizing glycemic control, pre-surgical showers, and skin antisepsis with agents like chlorhexidine-gluconate [6]. The impact of each of these practices, including topical chlorhexidine-gluconate application, on surgical site infection rates warrants further investigation. According to the global safe surgery protocol, implementing preventable care bundles is a complex process requiring multi-disciplinary collaboration [17,18]. Recent studies have shown that communication inconsistencies frequently occur in the operating room, leading to a persistent increase in surgical site infections [19,20]. Insufficient communication between the theater nurse and surgeon may be attributed to the quality of teamwork between nurses being less proficient than what surgeons expect [21]. Surgical site infection prevention involves striking a balance among costs, disability and loss, and the priorities of patients [4]. Surgical patients interact with many healthcare professionals throughout their post-operative experience and rely heavily on operating room nurses to provide high-quality care that promotes positive outcomes, including the prevention of surgical site infections. Operating room nurses contribute significantly to surgical site infection prevention through both individual and collaborative practices. Their individual responsibilities include rigorous adherence to hand hygiene protocols, meticulous application of aseptic techniques during surgical procedures, administering prescribed prophylactic antibiotics, and correct donning of sterile surgical attire and personal protective equipment [22]. Furthermore, they contribute to maintaining a controlled operating room environment by minimizing unnecessary traffic and door openings. These individual practices are reinforced by collaborative efforts within the surgical team, including the utilization of surgical safety checklists to verify critical aspects of surgical site infection prevention prior to incision. This comprehensive, team-based approach ensures the maintenance of a sterile field and minimizes the risk of microbial transmission, ultimately contributing to patient safety. Nonetheless, qualitative studies exploring the viewpoint of theater nurses regarding intraoperative surgical site infection occurrences are scarce. Studies indicate that besides technical proficiency, theater nurses need skills such as efficient communication, being cognizant of their surroundings, and managing stress to guarantee safe care practices throughout the intraoperative phase [22,23]. Highly qualified nurses are required in the operating room for the duration of the peri-operative period [24,25]. The study aim of this study was to explore operating room nurses’ experiences and perceptions of surgical site infection prevention practices during the intraoperative phase, during the COVID-19 pandemic in Hong Kong. This study can help healthcare systems better prepare to support the needs of surgical patients during further public health crises.

## 2. Methods

### 2.1. Study Design

This study explored the lived experiences of operating room nurses in preventing intraoperative surgical site infections using a descriptive phenomenological approach. Semi-structured interviews facilitated an in-depth understanding of these experiences. Valuing the unique perspectives of operating room nurses, this study employed a phenomenological approach to understand their experiences with surgical site infection prevention. We sought to capture both shared and individual experiences and their impact on the healthcare system. This methodology allowed us to explore the emotional impact of surgical site infection prevention on operating room nurses during the COVID-19 pandemic, enriching the existing knowledge base in this critical area. To ensure high-quality reporting of the qualitative methods and findings, this study followed the Consolidated Criteria for Reporting Qualitative Research (COREQ) checklist [26].

Study participants were thoroughly briefed on the study details and subsequently provided informed consent before participating in interviews. Before the interview, participants received information about the study’s background, purpose, and the entire interview process. They were volunteers and were informed that they could withdraw from the study without any negative consequences. To ensure confidentiality and anonymity, the participants’ data were encrypted and were only accessible to members of the research team. The Research and Ethics Committee at Caritas Institute of Higher Education approved the study (HRE210111), confirming its adherence to the ethical principles of the Declaration of Helsinki.

### 2.2. Participants

Using purposive sampling, this study recruited 21 nurses working in operating rooms at Hong Kong acute hospitals, each with at least 2 years of professional experience. Recognizing the constraints of the ongoing COVID-19 pandemic and the pivotal role of our participants, interviews were conducted remotely using telephone or digital platforms like Zoom. During the COVID-19 pandemic, this approach enabled comprehensive data collection while prioritizing participant safety, minimizing service disruptions, and adhering to public health guidelines.

### 2.3. Data Collection

Data collection began with cluster managers contacting potential participants after being informed about the study’s aims and methodology. Following this, researchers independently coordinated with interested theater nurses who had expressed their willingness to participate. Data collection took place between December 2021 and May 2022. Interviews were conducted in Chinese using a password-protected Zoom environment (Nasdaq, San Jose, CA, USA), scheduled at times convenient for participants. Each interview, lasting approximately 40 to 60 min, followed a consistent interview guide to maintain data collection consistency. During these sessions, researchers also took detailed field notes to capture contextual nuances for subsequent analysis. The structured questions used in the interview guide are presented in Table 1. Post-interview, the dialogues were transcribed verbatim. Participants were informed they could request adjustments to or copies of their transcripts, though no such requests were made. To validate the data, the interview questions were scrutinized by a panel composed of a qualitative scholar and an operating room nursing specialist, and to confirm alignment with the aim and focus of this research study. All participants provided written informed consent for their interviews to be audio-recorded. It is important to note that the researchers involved in conducting the interviews were volunteers, with the freedom to participate or withdraw from the study as desired. Data saturation, the point at which further data collection ceased to yield new insights relevant to the research question, was achieved in this study. Specifically, after the 19th interview, analysis of the subsequent interviews confirmed the absence of novel concepts, themes, or perspectives related to the study’s aims. This redundancy of information validated the achievement of data saturation and justified the conclusion of data collection.

### 2.4. Analysis Strategy

A descriptive phenomenological approach, guided by Colazzi’s (1978) method, was employed to analyze the data pertaining to healthcare providers’ experiences with surgical site infection prevention in the operating room [27]. This methodological choice was predicated on its established efficacy in elucidating the nuanced meanings individuals attribute to their lived experiences, a critical aspect of understanding the complexities intrinsic in surgical site infection prevention. Colazzi’s seven-step process provided a systematic framework for analyzing detailed descriptions and field notes, facilitating the identification of significant statements, the distillation of their intrinsic meanings, and the subsequent synthesis of a comprehensive understanding of the phenomenon [27]. This rigorous approach enabled the researchers to move beyond superficial interpretations and delve into the essence of healthcare providers’ lived experiences concerning surgical site infection within the dynamic and demanding context of the operating room. The NVivo software (NVivo Version 12, QSR International, Burlington, MA, USA) was employed for systematic data organization after each interview session. Two researchers (J.Y. and Z.T.) meticulously transcribed the recordings of each interview. This seven-step strategy identifies developing patterns by (1) gaining an understanding of the collected data, (2) finding meaningful descriptions, (3) applying reflective bracketing to shape interpretations, (4) identifying the key themes, (5) constructing a comprehensive description, (6) delineating the fundamental structure of the phenomenon, and (7) ascertaining the validity of the comprehensive depiction and underlying structure [27].

Every recorded interview was transcribed word-by-word and analyzed [27]. Two researchers (A.Y. and K.M.R.Y.) independently scrutinized all the interview content, emphasized notable statements, and determined the underlying themes. The research team monitored all analyzed data and compared the sub-themes, identifying similarities. All research team members discussed the analysis of the results to reach a consensus. Lincoln and Guba’s demanding standards ensured the rigor and trustworthiness of the research [28,29,30], including member checking after the interviews, auditing the data analysis process, engaging in reflexive discussion among the researchers to assess the psychological feelings of participants, and purposeful sampling of participants from different acute hospitals to capture extensive perspectives and experiences [31,32,33,34,35] (Supplementary Table S1). The final themes were derived through consensus and are detailed in the results.

## 3. Results

Twenty-one nurses working in operating rooms from acute hospitals were invited to undertake qualitative interviews. They agreed to participate in individual interviews on their views on surgical site infection prevention in theater nursing care in Hong Kong. Our recruitment included eight male and 13 female nurses, aged between 20 and 50 years, with a median age of 36 years. All participants had at least 2 years of experience in operating room nursing care-related occupations throughout the COVID-19 pandemic, with their experience ranging from 2 to 21 years and averaging approximately 8.3 years. The demographics and characteristics of the participants are listed in Table 2.

The essence of the phenomenon gathered from the study results was that “little things make a big difference”, according to the experiences of nurses in preventing surgical site infections during the intraoperative stage in the operating room. Four themes emerged from the interviews: (a) ensuring safety and minimizing threats, (b) facing silent, intangible, and hidden risks, (c) team collaboration in eliminating risks, and (d) persistent knowledge acquisition.

### 3.1. Theme 1: Ensuring Safety and Minimizing Threats

There are various perspectives among different healthcare professions, but multi-disciplinary collaboration can build a strong support system to safeguard patients against any potential threats. Participants reported that some colleagues needed to build trust among themselves to establish a sense of confidence in the professional skills of the team. In the operating room, it is not easy to determine which views should be prioritized in intraoperative care. However, ensuring safety and minimizing threats are vital components of surgical site infection prevention and patient care.

Most participants reflected that alongside multi-disciplinary collaboration, members of surgical teams develop a sense of teamwork by aligning their professional skills and ensuring stable collaboration to reduce any potential risks of surgical site infection. Mutual trust among team members can enhance their strengths, professional knowledge, and skills, and this can help them modify their workplace behavior in the interest of patient safety. Although several participants expressed that they sometimes deal with difficult decisions and modifications during an operation or surgical procedure, the mutual trust among surgical team members influenced the decision-making process. A confident surgical team has the ability to efficiently cope with any unexpected risky situations.

*“Every hospital has its own strategic training method for nurses in operating room depending on the operating room. When surgeons carry out surgical procedures, they would rather work with theater nurses and anesthetists whom they know and those who are familiar with the surgical procedures. This depends on communication among the staff: nurses in operating rooms work better with some people than with others”.*

(P06)

Some participants stated that some of their colleagues were reluctant to collaborate with other healthcare team members, causing friction and conflict. This dysfunctional work environment affects the safety of patients. Team members of different ranks must ensure the competence of their fellow professionals before carrying out surgical procedures to ensure a safe surgical environment. Moreover, every colleague must be willing to listen to constructive feedback in relation to the prevention of surgical site infections.

“*Some new junior colleagues are not familiar with the working environment of the ORs. They are afraid of certain surgeons and have communication difficulties. They become nervous and frustrated in this situation. Some workers have even attempted to resign or transfer surgical units as this situation has affected their work. At times, insecurities may develop, and errors may occur. For example, one junior nurse collaborated with an orthopedic surgeon as a scrub nurse to perform a total hip arthroplasty, but the junior nurse felt that the orthopedic surgeon was very intimidating, which made her nervous and prone to making mistakes. She placed the short raytex (surgical swabs) that she used to clean the instruments into the open wound where the implant had been installed during the total hip arthroplasty process*”.

(P08)

Some participants contemplated their apprehensions and experimented with various approaches to maintain a secure work setting for their colleagues while minimizing the possible hazards of surgical site infections.

“*The cleaning of the entire operating room is the responsibility of the outsourced staff of ISS (Integrated Service Solutions Facility Services Limited), but they are generally older and less educated. In order to ensure a clean environment in the operating room, surgeons and nurses are usually present. I feel a strong responsibility to maintain a clean and safe operating room environment. I prioritize clear and accessible communication when guiding eternal cleaning staff, using visual aids like simplified Chinese characters, images, and photos to ensure everyone understands the cleaning procedures, regardless of their educational background or language proficiency. This approach helps facilitate effective collaboration and ensures a consistently clean and safe surgical space*”.

(P07)

### 3.2. Theme 2: Facing Silent, Intangible, and Hidden Risks

Healthcare teams working in ORs maintain their professional responsibilities to ensure and monitor the safety of patients during surgical procedures and operations. Providing a sterile field with no contamination is essential for creating an optimal surgical environment. Theater nurses apply their specialized expertise to assess the correlation between the risk of surgical site infections and the existence of microorganisms. Other colleagues may have to adapt to recognize the hidden risks and threats of microorganisms in the operating room. Therefore, all healthcare teams must pay attention to getting rid of microorganisms during the intraoperative stage in the operating room and thus prevent surgical site infections. One participant who had over 10 years of experience working in operating rooms reported that she adhered to aseptic techniques regarding hygiene in the operating room, as follows:

“*I know the sterilization and aseptic technique of the current concept in operating rooms are very important, but surgeons and nurses have very different opinions regarding the concept of aseptic technique. In the operating room, some surgeons do not pay much attention to aseptic techniques. They (surgeons) lack collaboration with the surgical team in the operating rooms. I often need to tell new colleagues or surgeons that they must wear sterile gloves, or they need to adjust the surgical gown. Few surgeons do not follow proper surgical hand washing before the operation. On one occasion, the surgeon put on the sterile gown in the middle of the operation, and he used his own elbow to hold his glasses … when some nursing students had clinical attachments here, they walked too close to the sterile field. I had to remind all of them (surgeons, nursing students, and healthcare workers) that they must enter the operation theater through double doors. I always monitor all the things that happen in the operating room environment to give the surgeon an overview, control the flow of people, and at the same time focus my attention on what is happening during the surgical operation*”.

(P02)

Although there are guidelines to prevent surgical site infections, operating rooms seldom report the occurrence of surgical site infections after surgical procedures performed by colleagues. The lack of feedback on preventing surgical site infections between healthcare teams in operating rooms leads to conflicts between healthcare professionals in reinforcing routines and strategies to prevent surgical site infections. It is difficult to assess and explore the hidden risks. The lack of evidence on useful routines is a safety risk and can undermine surgical site infection prevention efforts. Therefore, most participants were hesitant and paid attention to the essential concept of aseptic techniques, owing to the lack of evidence at their institutions. Participants insisted on developing a fundamental ground, with a strong desire to prevent surgical site infections and cope with the hidden dangers of surgical procedures. All participants shared the mission to establish a protected safety zone in their workplace.

“*The operating room in each hospital has its own culture. The staff of each rank sticks to their own duties, does their own part and checks, and knows their role and responsibility. Therefore, for the prevention of surgical site infections in the operating room, on-site nursing educational training provided to staff of all ranks, cooperation between colleagues, and knowledge of patient education is very important*”.

(P15)

### 3.3. Theme 3: Team Collaboration in Eliminating Risks

Especially during the challenging period of the COVID-19 pandemic, surgeons, as leaders of the surgical team, played a crucial role in fostering heightened awareness of surgical site infection prevention. Their leadership in promoting accurate adherence to infection control protocols, coupled with a strong emphasis on team collaboration, proved essential in reducing surgical site infection risks within the operating room environment. This period indicated the critical importance of a unified team approach to risk elimination in the face of heightened infection control challenges.

Operating room units follow a traditional hierarchical organizational structure to ensure stability and safety. All participants work under stressful conditions and must manage unexpected surgical situations where this traditional pattern helps to diffuse the pressure and provide a more comfortable and relaxed atmosphere. However, this working atmosphere can sometimes jeopardize the ability of the staff to function as a healthcare team. Some participants expressed that those surgeons, who are at the top positions in the surgical team, made them feel unimportant and minimized their contribution. Theater nurses felt that the prevention of surgical site infections was often ignored by surgeons and other surgical team members.

“*I go to work every day and find no one is interested in the prevention of surgical site infections. However, I will not tire of reminding myself that it is quite unfortunate for patients to undergo operations. If the operation fails due to surgical site infections, patients will stay in the hospital for a longer period. Due to the COVID-19 pandemic, visitors are not allowed in the ward. I empathize with patients who feel miserable and lonely. Therefore, I cannot give up on what I think is correct (prevention of surgical site infections). If I do not adhere to these principles of aseptic technique and prevention of surgical site infections, patients will suffer more. If the entire operating room’s team follows the same correct principles in collaboration with each other, the risk of surgical site infections will be eliminated in operating rooms*”.

(P11)

All participants expressed that collaboration in the operating room is very important to build an optimal surgical environment, which allows the nurses to become confident in eliminating the risks of surgical site infections. They also emphasized that strengthening the confidence of theater nurses is key to preventing surgical site infections. Experienced theater nurses are recognized by the surgical team, and they demonstrate their professional competency by using skills that reduce the risks of surgical site infections during the COVID-19 pandemic. Their role safeguards every colleague in the operating room by ensuring adherence to hygiene guidelines and operating room routines.

“*During the challenging COVID-19 pandemic in Hong Kong, when anxieties were understandably high, the importance of multidisciplinary collaboration in the operating room became even more pronounced. The success of a surgical procedure relies on the collective efforts of the entire team, not solely on the surgeon. Effective collaboration between surgical teams and nurses was most important to upholding aseptic principles and preventing both surgical site infections and the spread of the coronavirus. To ensure patient safety and address these critical concerns, I prioritized providing comprehensive on-site training to colleagues, encompassing aseptic techniques, surgical site infection prevention, and COVID-19 infection prevention measures, including pre-duty rapid screening tests. This collaborative, multi-faceted approach was essential for handle the complexities of the pandemic and maintaining a safe surgical environment*”.

(P21)

### 3.4. Theme 4: Persistent Knowledge Acquisition

Participants underscored the significance of preparedness and possessing the capacity to adjust and conduct themselves professionally when collaborating with surgeons and anesthesiologists in the operating room in surgical site infection prevention and patient care. Additionally, the experiences of participants highlighted the value of embracing adaptability and receptiveness to change.

Based on the participants’ interviews, embracing alterations, novel procedures, emerging evidence, and their subsequent impacts is of paramount importance, even if it presents challenges for theater nurses. Furthermore, the narratives accentuated the participants’ belief in the significance of each individual executing their designated role as intended, thereby preventing unforeseen deviations from transpiring in the operating room.

“*I have come to appreciate the importance of being receptive to change and adapting to new procedures and their impacts. I have faced challenging moments when embracing these alterations, but I learned that such adaptability is crucial for the betterment of patient care and the growth of my own capabilities. I have witnessed firsthand how a well-coordinated team can create a safe and efficient environment, ultimately leading to better patient outcomes. By nurturing a strong sense of responsibility and commitment among my colleagues, we have been able to foster a harmonious and supportive atmosphere that enhances our collective skills and sensibilities*”.

(P05)

Participants portrayed their duties in the operating room as complex and demanding. Most participants indicated their wish to be extensively prepared and to devote sufficient time to carrying out their responsibilities to the highest level of proficiency. Moreover, they stressed the value of adaptability when working alongside surgeons and anesthesiologists within the operating room setting.

“*I can be quite intricate and demanding. For me, it is essential to be extensively prepared and allocate enough time to carry out my responsibilities to the best of my abilities, and it has been a key factor in ensuring the highest level of patient care. I recognize the importance of being adaptable when working alongside Dàfū (surgeons) and anes (anesthesiologists). Flexibility in collaboration helps me maintain a smooth workflow, creating a more efficient and effective environment for our team*”.

(P16)

## 4. Discussion

This study helps to explore the experiences of nurses working in the operating room in the intraoperative prevention of surgical site infections. By uncovering real-world challenges and strategies, we sought to generate insight with the potential to improve practices within surgical settings. Preventing surgical site infections requires a strong team approach. Success depends not only on overall team performance but also on individual dedication and organizational support. A culture of shared responsibility and empowerment is essential. The finding of this study explores effective team activities and shared responsibilities, like communication and cross-training, to optimize team performance and improve patient outcomes. Moreover, individual and organizational legitimacy have an intricate relationship in care settings, especially in the operating room. It is the responsibility of the theater nurses to ensure an ideal hygienic setting inside the operating room to prevent surgical site infections throughout the intraoperative stage. Nevertheless, some nurses experience annoyance and occasionally resign due to this responsibility. The whole operating room team must communicate to establish an ideal setting to prevent surgical site infections. In operating room units, different members of the team sometimes have different priorities than establishing proper prevention procedures for surgical site infections, resulting in the nurses feeling that their contributions are not appreciated or taken seriously. These variances in viewpoint among the professionals indicate teamwork-related problems. Studies revealed that nurses frequently have difficulties in addressing arguments, clarifying misconceptions, and pointing out errors [34,36]. These problems are frequently the result of sex- or hierarchy-related barriers. Some theater nurses in operating rooms experience mental stress throughout the pre-operative and intraoperative stages [37,38]. The constant stress, as a consequence of inadequate communication and judgment in the operating room, affects their surgical performance [39,40]. Findings from this study indicate that seasoned theater nurses, regarded as authoritative figures by fellow operating room team members, are better equipped to manage challenging situations arising during surgical procedures compared to their less-experienced counterparts. Nurses require more assistance and support when discussing their concerns within the operating room setting [37,38]. Therefore, developing and establishing a supportive work setting will help nurses better prevent surgical site infections and improve patient care.

A crucial responsibility of the nurse working in the operating room is to ensure the prevention of surgical site infections. However, evidence from various care loads and services is unclear. Surgeons may be inclined to bypass rules and written guidelines while nurses attempt to equate following rules and written guidelines with professionalism [41,42,43]. Therefore, it is challenging for operating room team members to successfully form a cohesive unit. Study participants conveyed that other colleagues do not place a high priority on adhering to the measures designed to prevent surgical site infections, an attitude that makes it difficult to establish an ideal surgical setting. Some studies have emphasized the underrated outcome of care bundles [44,45]. Prevention of surgical site infections requires an awareness of the influence of distinct competencies of the various staff members [46,47]. Improved clinical practice, judgment, and specialized education for healthcare providers are linked to better patient outcomes [25,48]. This suggests that targeted training may reduce surgical site infections. However, strong evidence, like studies showing a direct link between specific training and lower surgical site infection rates, is significant. Further research should explore healthcare providers’ clinical judgment and weigh contributing factors to optimize surgical site infection prevention strategies and support healthcare providers effectively [25,48].

Additionally, this study provides essential evidence on the association between the risk of surgical site infections and the functionality of an operating room team. Sufficient time is needed to establish a working team. Nowadays, the intricacy of contemporary surgery requires teams to be particularly reliable, experienced, self-confident, and competent in their individual roles. These contemporary operating room teams must establish collaborative interrelationships with each other to remain cohesive for long durations and enhance patient outcomes [32,36]. Conflicts among teams may influence patient safety, such as the prevention of surgical site infections. Thus, operating room management guidelines should focus on minimizing conflicts and improving the work environment to ensure that collaborative and open-minded teams are formed. According to Kuy and Romero, collaboration is an important component for all members of the operating room team, and management should conduct training to ensure collaborative teamwork [49]. On the contrary, teams in which the members collaborate only to achieve their objectives are often unsuccessful [33,34]. These kinds of teams elevate the risk of surgical site infections given the lack of trust, reliance, and appreciation of the skills of their colleagues.

Nurses significantly contribute to preventing surgical site infections within the operating room [35,50]. Owing to the rapid development of surgical methods, nurses need to continuously increase their competencies in operating room procedures [47,51]. The present findings demonstrate that cohesive teams with specified competencies and high collaboration are keys to preventing surgical site infections. To ensure peri-operative safety and enhance patient outcomes, all aspects of teamwork should be strengthened [52,53]. Global healthcare provider shortages and high turnover disrupt stable team development necessary for safe peri-operative care; constant onboarding hinders the cohesive teamwork and shared understanding gained through long-term collaboration, potentially compromising patient safety [54,55]. In addition, collaborative support and lack of restriction can nurture an excellent multi-disciplinary team [37,55].

Studies have shown that organizational determinants have a large influence on how healthcare professionals provide peri-operative care and ensure patient safety [56,57]. Operating room team members often encounter challenges in finding opportunities and platforms to establish and work towards shared goals due to conflicts with their unit heads. Hence, organizational-level efforts are required to ensure that the operating room team works as a cohesive unit. It is crucial to recognize and support the vital role of nurses in preventing surgical site infections in the intraoperative stage. If the prevention of surgical site infections is approached with a strong focus on justifiability, nurses will continue to be recognized as vital professionals in the operating room.

The findings of this study emphasize the crucial role of team collaboration in preventing surgical site infections while concurrently exposing hierarchical barriers within operating room teams, particularly impacting nurse–surgeon dynamics [37,55]. These power imbalances can inhibit crucial information sharing, and potentially endanger patient safety. Alleviating these challenges requires a multifaceted approach confining organizational culture and leadership [54,55]. Cultivating shared responsibility and mutual respect is most important and achievable through empowering nurses in surgical site infection prevention decision-making. Implementing structured communication protocols, such as checklists or briefings, facilitates clear information exchange [41,42,43]. Furthermore, fostering open communication, where nurses feel safe expressing concerns, is essential. Transformational leadership, characterized by open communication and shared decision-making, is crucial for dismantling hierarchical barriers. Promoting psychological safety, where all team members feel valued, further enhances communication. Finally, organizational initiatives like interprofessional training and simulation exercises provide opportunities for practicing collaborative skills, ultimately optimizing surgical site infection prevention and patient outcomes [37,55].

These study findings emphasized the importance of allocating sufficient time for preparations. To foster the growth of clinical competence among junior colleagues, support from team members should be offered. Knowledge is acquired through practical clinical experiences and engagement with various medical disciplines [58,59]. In this context, theater nurses articulated the necessity of possessing diverse forms of expertise to maintain competence and technical proficiency. Moreover, they stressed the significance of staying current with cutting-edge innovations to adapt to the constantly shifting realm of surgical procedures [55,59]. The findings of this study highlighted that nurses believe basic rules regarding theater behaviors, equipment, clothing, and procedural practices require continuous monitoring, evaluation, and reinforcement. This constant vigilance is essential not only for maintaining current standards but also for integrating new technologies and evolving best practices into daily routines. This emphasis on continuous learning and adaptation highlights the dynamic nature of the surgical environment and the nurses’ commitment to providing safe and effective patient care. The implications of this study cut across multiple facets of healthcare. It highlights the role of nurses and the importance of teamwork, ongoing skill development, and clear communication in reducing surgical site infections. Also, it advocates for policy changes, including the provision of regular training, effective communication systems, the inclusion of non-technical skills in nursing education, and the development of public health strategies to manage the societal and economic burdens of surgical site infections.

### 4.1. Limitations

This study has limitations. The Hong Kong-centric participant pool, drawn solely from acute care hospitals, limits generalizability to other settings like ambulatory surgical centers or rural hospitals. Future research should diversify participant recruitment to enhance external validity. Reliance on self-reported data introduces potential social desirability bias, influencing participant responses. Mitigating this requires incorporating observational data or validated instruments in future studies. While acknowledging heightened infection control awareness due to COVID-19, the study does not fully explore its impact on operating room dynamics and nursing roles. Future research should investigate pandemic-induced changes in hierarchies, communication, and resource allocation, and their effects on surgical site infection prevention. This could involve comparative pre- and post-pandemic studies or exploring nurses’ experiences with altered roles during the pandemic. Despite these limitations, the study’s phenomenological approach provides valuable insights into Hong Kong nurses’ experiences, laying the groundwork for future research.

### 4.2. Implications for Practice

Conducted during the COVID-19 pandemic, this study’s findings may reflect the exceptional circumstances and adaptations characterizing this period. The pandemic’s influence on staffing, protocols, and healthcare system capacity likely shaped participant experiences and perspectives. Subsequent research should address these limitations by investigating the enduring impact of the pandemic on theater nurses, including potential shifts in practices, attitudes, and perceptions; investigating how the pandemic has reshaped these is significant for informing future training, resource allocation, and support systems. Specifically, examining the pandemic’s influence on communication dynamics, teamwork efficacy, and occupational stress levels within peri-operative teams warrants further investigation.

## 5. Conclusions

This study emphasizes the complex and multi-faceted role of nurses in preventing surgical site infections within the operating room. Establishing collaborative forums for quality improvement, recognizing, and valuing the expertise of nurses, and fostering stable team structures are essential for effective surgical site infection prevention. Continued caution in surgical site infection prevention is important for ensuring patient safety intraoperatively, particularly given the increasing threat of antibiotic resistance. Maintaining a high priority on operating room safety, including minimizing bacterial load throughout the surgical environment, is crucial. This research highlights the importance of reciprocal knowledge exchange and attentiveness among operating room team members, especially during critical periods like the COVID-19 pandemic. Furthermore, a reassessment of traditional hierarchical structures within the operating room is warranted to align with the evolving complexities of contemporary surgical practices. This will empower nurses and other team members to contribute their expertise effectively, ultimately enhancing patient safety and optimizing surgical outcomes.

## Figures and Tables

**Table 1:** Interview guide.

No.	Probing Questions
1.	Can you describe your daily work in your profession and your working situation in the operating rooms setting during the COVID-19 pandemic?
2.	Can you tell me about what surgical site infection prevention means to you in your daily work as a nurse working in operating room?
3.	What has your surgical site infection prevention experience been like in operating rooms? Do you have an example during the COVID-19 pandemic?
4.	What do you do to prevent surgical site infections during the intraoperative phase in operating rooms? Do you have an example during the COVID-19 pandemic?
5.	What difficulties or challenges did you face with intraoperative surgical site infection prevention in your daily work in operating rooms? Do you have an example amidst the COVID-19 pandemic?
6.	How have the COVID-19 pandemic and the ongoing human capital deficit impacted your thoughts, feelings, and practices related to surgical site infection prevention in the operating room, specifically regarding changes in procedures, modalities, and challenges in maintaining best practices?

**Table 2:** Demographic data of participants in operating rooms.

Characteristic	All		Male Nurses		Female Nurses	
	*n* = 21		*n* = 8		*n* = 13	
	*n*	%	*n*	%	*n*	%
Age						
20–30	5	23.8	3	37.5	2	15.5
31–40	10	47.6	1	12.5	9	69
41–50	6	28.6	4	50	2	15.5
Marriage and children						
Single	10	47.6	2	25	8	61.5
Married	10	47.6	6	75	4	30.8
Divorced	1	4.8	0	0	1	7.7
Education level						
Higher Diploma	2	9.5	1	12.5	1	8
Bachelor’s degree	9	42.9	3	37.5	6	46
Master’s degree	10	47.6	4	50	6	46
Current position						
Enrolled nurse	2	9.5	1	12.5	1	8
Registered nurse	14	66.7	5	62.5	9	69
Advanced practice nurse	5	23.8	2	25	3	23
Number of year(s) of working/experience in operating rooms						
2–6	10	47.6	4	50	6	46
7–11	5	23.8	0	0	5	39
12–16	5	23.8	3	37.5	2	15
17–21	1	4.8	1	12.5	0	0
Religious beliefs						
None	11	52	6	75	5	38
Christian	9	43	2	25	7	54
Buddhist	1	5	0	0	1	8

## Data Availability

The data that support the findings of this study are available from the corresponding author upon reasonable request.

## Supplementary Materials

The following supporting information can be downloaded at: https://www.mdpi.com/article/10.3390/healthcare13060579/s1, Table S1: Summary of Strategies Used to Ensure Trustworthiness.
