# Supplementary material for: The Impact of COVID-19 on Healthcare Services, Risk Management, and Infection Prevention in Surgical Settings: A Qualitative Study
Source: Healthcare (Basel). 2025 Mar 7;13(6):579. doi: 10.3390/healthcare13060579 (PMC11942026; doi:10.3390/healthcare13060579)
Supplement: Supplementary file 1 [file healthcare-13-00579-s001.zip › healthcare-3470251-supplementary.pdf]

Supplementary Table S1. Summary of Strategies Used to Ensure Trustworthiness.

Trustworthiness criteria	Intention	Approaches for Ensuring Trustworthiness	Implementation Notes
Credibility	To inspire faith that the results are true, credible, and believable	Prolonged and diversified engagement with each composition	- Due to the COVID-19 pandemic at the time of the research, it was not possible to conduct participant observation in the field. The Government of Hong Kong Special Administrative Region implemented a policy restricting multi-household gatherings in private premises to no more than two households or groups in the community. The objective of this policy was to curb the transmission of the virus within the community. - Nevertheless, participants from various operating rooms were recruited through nursing administration departments that maintain regular communication about clinical research with the authors' affiliated institutions.
		Conducting interviews using comprehensive methods and techniques	- After obtaining ethical approval, the interview guide was tested at three induction meetings; three pilot interviews were then conducted via Zoom. The data obtained from these interviews were included in the final data analysis.
		Establishing the authority of the investigators	- All members of the research team possessed the necessary knowledge, data management skills, and practical experience in qualitative research, with no less than four years of experience in their respective roles.
		Collecting all materials that ensure referential adequacy	- Field notes were utilized to document contextual information provided by participants to ensure accurate data analysis. Additionally, the field notes were analyzed alongside the transcripts.
		Regularly conducting peer debriefing sessions	-The research team held regular debriefing sessions every two weeks with Fellows from the Hong Kong Academy of Nursing & Midwifry to verify that there were no underlying biases, perspectives, or assumptions on the part of the researchers.
Dependability	To ensure the reproducibility of the findings of this qualitative inquiry within the same cohort	Wealthy description of study methods	- The research papers provided a detailed and clear description of the study methods.
		Set up an audit trail	- To create a comprehensive record of the data collection process by all researchers. - Member checking was achieved to ensure the accuracy of the interpretations derived from the participants, thereby reinforcing the credibility of their accounts.
		Progressive replication of data	- To assess the accuracy of coding and the reliability of the coders throughout the data analysis process by all researchers.

Confirmability	To enhance the assurance that the results would be validated by other researchers	Reflexivity	- Methods such as reflexive journals and weekly investigator meetings were implemented.
Transferability	To enhance the generalizability of the results for application in diverse contexts or situations	Data saturation<br>Thorough depiction	- Data saturation was deemed to have been achieved when no new themes emerged from the participants. A consensus was reached among all researchers regarding the attainment of data saturation.<br>- The participants provided extensive descriptions in their quotes, enabling a contextual explanation of the meanings behind their affirmations.
